# Supplementary material for: Food Protein Sterylation: Chemical Reactions between Reactive Amino Acids and Sterol Oxidation Products under Food Processing Conditions
Source: Foods. 2020 Dec 17;9(12):1882. doi: 10.3390/foods9121882 (PMC7766307; doi:10.3390/foods9121882)
Supplement: Supplementary file 1 [file foods-09-01882-s001.pdf]

foods

MDPI

Supplementary Files.

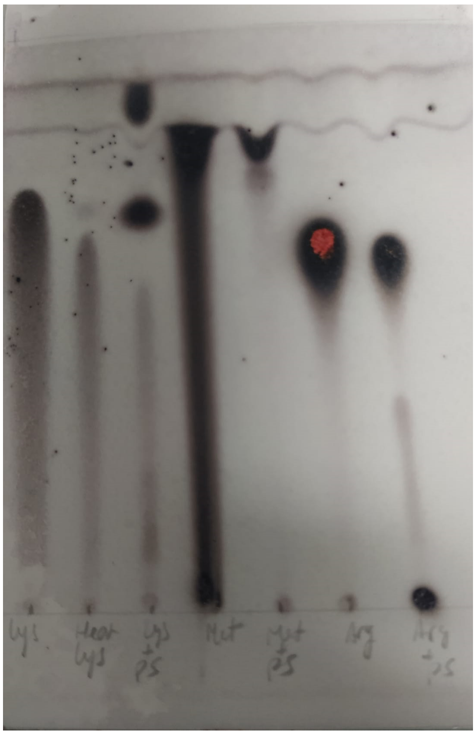

Figure S1. Thin Layer Chromatography (TLC) of reaction models between protected reactive amino acids (N_{α}-Boc-arginine, N_{α}-Boc-lysine and N_{α}-Boc-methionine) and oxysterol (cholesterol $5\alpha,6\alpha$-epoxide). Incubation was performed at 160 °C for 60 min. Spot separation was observed spot separations were observed from mixture models containing cholesterol $5\alpha,6\alpha$-epoxide with N_{α}-Boc-l-lysine. From left to right: N_{α}-Boc-lysine (T0), Heated N_{α}-Boc-lysine (160°C for 60 min), N_{α}-Boc-lysine + cholesterol $5\alpha,6\alpha$-epoxide, N_{α}-Methionine, N_{α}-Methionine + cholesterol $5\alpha,6\alpha$-epoxide, N_{α}-Arginine, N_{α}-Arginine + cholesterol $5\alpha,6\alpha$-epoxide.

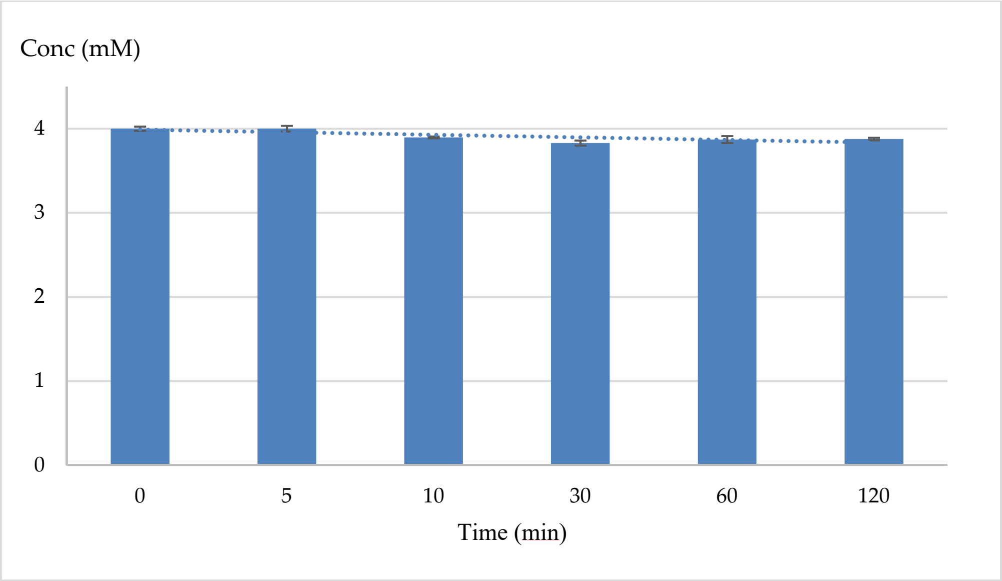

Figure S2. Levels of cholesterol $5\alpha,6\alpha$-epoxide in blank samples over the incubation time at 180°C.
